# A Structural Model of Emotional Intelligence, Resilience, and Stress in University Students

**DOI:** 10.3390/bs15070894

**Published:** 2025-06-30

**Authors:** Raquel Gilar-Corbi, Andrea Izquierdo, Juan-Luis Castejón

**Affiliations:** Department of Developmental Psychology and Didactics, University of Alicante, 03080 Alicante, Spain; raquel.gilar@ua.es (R.G.-C.); jl.castejon@ua.es (J.-L.C.)

**Keywords:** emotional intelligence, resilience, stress, structural equation analysis, university students

## Abstract

Mental health is an increasing concern, particularly among the younger population. Given that university life is a critical stage in the personal and academic development of some young individuals, identifying the factors that contribute to their well-being is essential. This study focused on exploring the role of emotional intelligence and resilience in the perceived stress of a sample of 338 university students from the University of Alicante. To this end, a structural equation analysis was used to evaluate the direct and indirect effects of the variables included in the model. The instruments used were the Trait Meta-Mood Scale, the Connor–Davidson Resilience Scale, and the Perceived Stress Scale. Based on the dimensions of the cascading model of emotional intelligence, the results indicate that emotional attention had a direct and positive effect on stress, while both emotional clarity and emotional repair influenced resilience directly and positively. In turn, resilience had a direct and negative effect on stress, acting as a mediator between the dimensions of emotional intelligence and stress. The final proposed model explained 49.3% of the variance in stress. This study provides empirical evidence regarding how these variables interact and underscores the importance of emotional abilities in stress regulation in the university setting.

## 1. Introduction

### 1.1. Stress in the University Stage

The university stage represents a crucial period in personal and academic development, characterized by notable transformations at the social, emotional, and cognitive levels. Most university students are in an age range that inherently involves notable life changes, making this stage a key moment for identity formation and the consolidation of interpersonal relationships (Campos-Ribeiro & Bolsoni-Silva, 2011). In this regard, Johnson (2018) noted an increase in mental health problems among young people aged 16 to 24, highlighting the need to address the factors influencing their well-being.

University offers an environment conducive to establishing multiple social interactions with teachers, peers, and new friends, and developing meaningful emotional relationships. These interactions play a fundamental role in acquiring social skills and emotional adjustment and are essential for facing the challenges of this stage successfully (Priestley et al., 2022). However, when these interactions are not managed appropriately, they can affect young people’s well-being and negatively impact their mental health (Zhao, 2024). According to Murali and Avudaiappan (2024), stress has been identified as one of the main threats to mental health in this group.

In addition to these social dynamics, academic demands can become notable sources of stress and, later, may lead to mental health issues. Thus, in addition to interpersonal conflicts, Murali and Avudaiappan (2024) identified high academic workload, economic difficulties, and procrastination as key stressors, all of which impair academic performance.

Although stress can manifest at all educational levels, its prevalence tends to increase at higher levels (Putwain, 2007). In this sense, Gutiérrez and Amador (2016) described stress as a particularly relevant mental health issue in the university context. Meanwhile, Harvanek et al. (2021) warned that high levels of stress may increase the risk of developing physical illnesses and psychiatric disorders.

Given this reality, it is essential to identify the risks and protective factors that impact the stress perceived by university students. Understanding which skills can act as stress buffers for students will enable the design of more effective strategies to promote their emotional adjustment. In this regard, possessing interpersonal skills not only facilitates the establishment of healthy relationships but also contributes to creating a positive learning environment based on respect and mutual understanding (Hernández-Perdomo et al., 2021).

Among these skills, emotional intelligence (EI) and resilience have been identified as key variables in stress regulation. In this regard, Torres-Montañez et al. (2023) found that people with higher levels of EI tend to employ more effective strategies to manage stress, particularly those focused on emotional regulation and problem-solving. In addition, in a recent systematic review, Jibaja-Balladares (2024) emphasized that high levels of resilience are associated with factors such as self-confidence, social and family support, psychological well-being, personal competencies, and optimism. Moreover, they are associated with better academic performance and reduced vulnerability to stress, anxiety, and depression. Therefore, exploring the role of these variables in the impact of stress is fundamental for designing interventions that promote the well-being of university students.

### 1.2. Relationships Between the Variables of This Study

One of the key factors in regulating social interactions and coping with stressful situations is EI. EI is defined as the ability to perceive, understand, and effectively regulate one’s emotions, in oneself and in others (Mayer & Salovey, 1997; Salovey & Mayer, 1990). These emotional skills directly influence the way individuals manage their interpersonal relationships and respond to emotionally demanding situations. In the university context, which is characterized by multiple academic and social pressures, EI can play a determining role in adaptation and coping, promoting better mental health and healthier interpersonal relationships (Agarwal et al., 2020).

The relationship between EI and stress has been widely documented in the university population. For example, García-Martínez et al. (2021), in a study of preservice teachers, found a negative correlation between the two variables, indicating that higher levels of EI are associated with lower levels of stress. Similar results were found by Aguilar-Priego et al. (2024) in another sample of university students. Similarly, Sánchez-Zafra et al. (2024) explored this relationship, highlighting that while emotional attention correlates positively with stress, emotional clarity and emotional repair correlate negatively with it. These findings suggest that not all components of EI impact stress in the same way.

Resilience, understood as the ability to adapt positively to adversity (Luthar et al., 2000) or cope with stressful situations effectively (Connor & Davidson, 2003; Hoge et al., 2007), is also presented as an essential resource in the university stage. Empirical evidence suggests a positive relationship between EI and resilience. This has been confirmed in studies by García-Martínez et al. (2022) and Rabal-Alonso and González-Romero (2023), who identified this association in preservice teachers. Similarly, Valverde-Janer et al. (2023) emphasized that emotional repair is a key component in strengthening resilience. These results have also been corroborated by other studies, such as those by Sojer et al. (2024) and Ang and Lau (2024), in various samples of university students.

Consistently with this relationship, the study by Cejudo et al. (2016), also conducted with university students, found that those with high levels of resilience and emotional repair reported higher life satisfaction. By contrast, higher scores in emotional attention were negatively associated with life satisfaction scores.

Resilience has been shown to have an inverse relationship with stress in the university population. Research by Litwic-Kaminska et al. (2023) and Ebenezer et al. (2020) corroborated that students with higher levels of resilience tend to experience lower levels of stress. This association was also found in the studies by Martínez-Cuevas et al. (2024) and Bukhari et al. (2023), who also found that greater resilience not only reduces stress but also impacts academic performance positively.

Some studies have analyzed the three variables of this study (EI, resilience, and stress) jointly in the university population. For example, Shin and Yeong (2013), in a study of nursing students, found significant correlations between all three variables, concluding that strengthening EI and resilience can contribute significantly to stress reduction.

Furthermore, although they incorporated other additional variables, Kökçam et al. (2022), Thomas and Zolkoski (2020), and Trigueros et al. (2020) agreed in noting a positive correlation between EI and resilience and a negative correlation between stress and both variables. In this regard, Hyeonsu and Mi-Ra (2020), who aimed to examine the antecedents of resilience, concluded that it is influenced by both EI and stress in university students.

### 1.3. This Study

While numerous studies have explored the relationship between EI, resilience, and stress in university populations, there remains a significant gap regarding the use of structural equation models to analyze these associations, with only a few studies having addressed these associations using structural equation models (e.g., Thomas & Zolkoski, 2020; Trigueros et al., 2020). Moreover, even fewer have placed a particular focus on the impact of EI and resilience on perceived stress, despite consistent scientific evidence suggesting that both variables act as protective factors against stress (e.g., Aguilar-Priego et al., 2024; Jibaja-Balladares, 2024; Kökçam et al., 2022). This study aims to fill that gap by applying a theoretically grounded cascading model that unpacks these complex relationships more precisely.

The interest in examining these variables among university students is justified by the increasing number of young people who enter university each year, making this a critical stage for analyzing their well-being. However, this study focuses on a particularly relevant sample: preservice teachers. Understanding how their emotional abilities interact is of particular importance as these future educators have the potential to transmit these abilities into their professional practice, influencing their future students and the teaching and learning processes they will lead (Calderón-Calderón, 2024; Cassullo & García, 2015).

The study most closely related to this one is that by Sarrionandia et al. (2018), which examined the relationship between EI, resilience, and perceived stress in university students, and found that resilience mediated the relationship between EI and stress, with EI acting as a negative predictor of perceived stress. However, this study used a total EI score, analyzing relationships only through observed variables. By contrast, this study uses a cascading model that allows for a more precise and theoretically grounded analysis of the relationships between the specific factors that make up EI. This approach is based on the “cascading model” proposed by Joseph and Newman (2010), grounded in theories of emotion, emotion regulation, and self-regulation (Gross, 2008; James et al., 2006). According to this model, the three EI dimensions are related in a specific order, as follows: emotion perception precedes emotional understanding, which, in turn, precedes emotion regulation. This approach offers a more detailed perspective on how the different components of EI relate to resilience and stress.

Thus, this aims to provide empirical evidence on the relationship between EI, resilience, and perceived stress in university students, using a structural equation modeling approach. This study contributes novel and methodologically advanced evidence that improves the understanding of the protective emotional and coping abilities essential not only for well-being during the university stage but also for the professional development of future educators, with potential implications for educational practices and student support programs.

### 1.4. Objectives and Hypotheses

The objective of this study was to investigate the influences of the dimensions of EI on resilience and stress, and the mediating role of resilience between the dimensions of EI and stress.

In addition, we set out to analyze whether the mediating effects of the EI dimensions (in cases wherein EI dimensions are mediating) follow the causal chain established by Joseph and Newman (2010) in their “cascading model”. This model establishes that the relationship between the three dimensions of EI is as follows: emotion perception precedes emotional understanding, which, in turn, precedes emotion regulation. In other words, the ability to understand emotions mediates the relationship between the ability to perceive emotions and the ability to regulate them, with the latter directly affecting resilience and stress.

**Figure 1:**
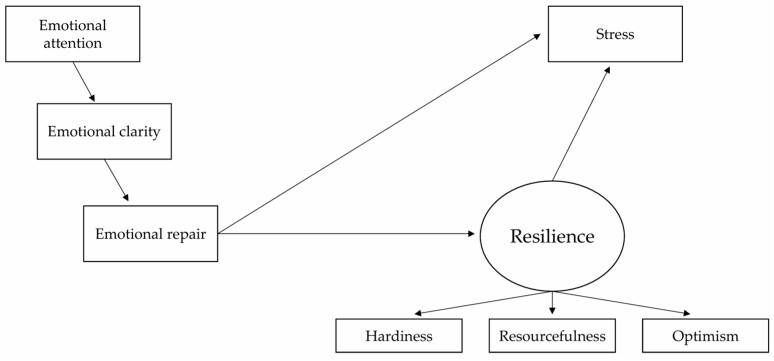
Hypothetical model (Model 1).

Thus, considering the connection between the dimensions of EI, and as shown in Figure 1, the initial model posits the following hypotheses.

**Hypothesis** **1.** *Emotional attention positively influences emotional clarity, which, in turn, positively affects emotional repair.*

**Hypothesis** **2.** *Emotional repair positively influences resilience and negatively influences stress.*

**Hypothesis** **3.** *Resilience mediates between emotional repair and stress, meaning that emotional repair positively influences resilience, and resilience negatively influences stress.*

## 2. Materials and Methods

### 2.1. Participants

This study included 338 students (27.2% male; 72.8% female) from the Faculty of Education at the University of Alicante (Spain). The average age of the participants was 20.55 years (SD = 4.173), with ages ranging from 17 to 50 years, and 93.2% of the students were between the ages of 17 and 24 years.

This sample (*n* = 338) ensures adequate effect size and power, since G*Power 3.1.9.7 (Faul et al., 2009) suggests that 146 participants are needed to detect an effect size of 0.15 with a power of 95% (alpha = 0.05, two-tailed) and 6 predictors. Soper’s (2024) online calculator suggests that 143 participants are needed to detect an effect size of 0.15 with a power of 95% (alpha = 0.05, two-tailed), with 1 latent factor and 7 observed variables.

### 2.2. Measures

#### 2.2.1. EI

EI was assessed using the Trait Meta-Mood Scale (TMMS-24) developed by Salovey et al. (1995) and adapted to Spanish by Fernández-Berrocal et al. (2004). This version comprises 24 items, organized into three dimensions, as follows: emotional attention, emotional clarity, and emotional repair. Fernández-Berrocal et al. (2004) reported Cronbach’s alpha coefficients of 0.90, 0.90, and 0.86 for these dimensions, respectively, indicating excellent internal consistency. Responses were recorded on a five-point Likert scale, ranging from 1 (strongly disagree) to 5 (strongly agree). An example of an item included in the scale is “I pay a lot of attention to how I feel” (item 8).

#### 2.2.2. Resilience

Resilience was assessed using the Connor–Davidson Resilience Scale (Connor & Davidson, 2003), adapted into Spanish by Manzano-García and Ayala (2013). This version comprises 25 items, organized into three dimensions: hardiness, resourcefulness, and optimism. The Cronbach’s alpha coefficients reported by Pozo-Rico et al. (2023) for each dimension demonstrated excellent internal consistency, as follows: 0.94 for hardiness, 0.93 for resourcefulness, and 0.92 for optimism. Responses were recorded on a five-point Likert scale, ranging from 0 (strongly disagree) to 4 (strongly agree). An example of an item included in the scale is “I give my best effort, no matter what” (item 10).

#### 2.2.3. Stress

Perceived stress was assessed using the Perceived Stress Scale by Cohen et al. (1983), which measures the emotional experiences and thoughts of participants over the previous month. The Spanish adaptation developed by Remor (2006) was used, which comprises 10 items. Remor (2006) reported a Cronbach’s alpha coefficient of 0.82, indicating good internal consistency. Responses were recorded on a five-point Likert scale, ranging from 0 (never) to 4 (very often). An example of an item included in the scale is “In the past month, how often have you felt confident about your ability to handle your personal problems?” (item 4).

To reduce the number of variables included in the model, the scores on the various factors of the EI and stress measures were taken as observed variables, while the resulting general factor was constructed from three factors (hardiness, resourcefulness, and optimism) and the resilience measure was used as a latent variable.

### 2.3. Procedure

The study received approval from the Ethics Committee of the University of Alicante (UA-2021-12-09_2), ensuring adherence to ethical principles in data handling and participant involvement.

Participant recruitment was carried out during regular class hours of compulsory courses in the Early Childhood and Primary Education programs at the Faculty of Education of the University of Alicante. All enrolled students were invited to participate, resulting in a final sample of 338 participants (44.47% of the total 760 students).

Before beginning the research, all participants were informed about the objectives and characteristics of this study and were assured of the confidentiality of their data. Participation was entirely voluntary, and informed consent was obtained before inclusion in this study. In addition, participants were given the option to withdraw from this study at any time.

After providing their consent, the participants completed the study questionnaires. The session lasted approximately one hour.

### 2.4. Design and Data Analysis

Based on the correlation matrix, structural equation analysis was employed using the maximum likelihood with robust standard errors (MLR) estimation method, which is useful in cases wherein the distribution deviates slightly from normality (Kyriazos & Poga-Kyriazou, 2023), to test the set of relationships hypothesized in the initial model. Thus, after identifying possible outliers, the fit of the initial model was tested, using as criteria the measures of absolute fit, χ^2^, goodness of fit index (GFI), standardized root mean square residual (SRMR), root mean square error of approximation (RMSEA), and the comparative fit index (CFI). For all of them, values of >0.95 and <0.06 for the RMSEA were established as the acceptance threshold (Byrne, 2006). The direct, indirect, and total effects obtained between the different variables included in the model were then analyzed. The statistical program JASP v0.19.3 (JASP Team, 2024) was used for all analyses.

## 3. Results

### 3.1. General Characteristics of Participants

Table 1 shows the frequency of males and females according to age groups. Pearson’s chi-square test was performed, and the results indicate that there were no significant differences between men and women in each of the age groups (χ^2^ = 1.351, df = 2, *p* = 0.509).

### 3.2. Descriptive and Correlational Analysis

Table 2 presents the means, standard deviations, and Pearson correlations among the study variables. Emotional clarity, emotional repair, and resourcefulness were the variables that showed significant positive correlations with all study factors, except for stress, with which they correlated negatively. Notably, emotional repair was strongly correlated with optimism (r = 0.552, *p* < 0.001). Stress, in turn, exhibited significant negative correlations with all factors except emotional clarity, with its strongest negative correlation observed with resourcefulness (r = −0.558, *p* < 0.001).

### 3.3. Structural Model Testing

Following the above, to strengthen the validity of the statistical model, a collinearity test was performed and good results were obtained. Taking stress as the dependent variable, the variance inflation factor (VIF) values ranged from 1.12 for emotional attention to 2.76 for hardiness—all less than 3—and the tolerance values were greater than 0.30 for all predictor variables. Cook’s distance values ranged from 0.000 to 0.033 (M = 0.003), all of which are appreciably less than 0.5. The Durbin–Watson statistic regarding the independence of errors reached the optimal value of 2.093.

To check the overall fit of the initial model, absolute and comparative fit indices were used (Table 3).

As can be seen in Table 3, the χ^2^ statistic is significant in all four models, indicating that the models do not perfectly fit the data. However, χ^2^ is very sensitive to sample size and may not be reliable for samples of >200 subjects (Bagozzi & Yi, 1988; Bollen, 1989); therefore, it is preferable to use alternative indices such as those indicated above.

In Model 1 (initial), CFI reached the value of 0.890, which is lower than the recommended 0.95 (Hu & Bentler, 1999). The RMSEA index is considered acceptable when it is in the range of 0.05–0.10 (Browne & Cudeck, 1989); in Model 1, its value was 0.148, exceeding the recommended limit. Similarly, an SRMR value between 0.05 and 0.08 indicates an acceptable fit (Kline, 2015); in this case, Model 1 presented a value of 0.095, which is slightly above the recommended threshold.

All regression coefficients in Model 1 were significant except the stress–repair regression coefficient, thus in Model 2, this relationship was eliminated.

**Figure 2:**
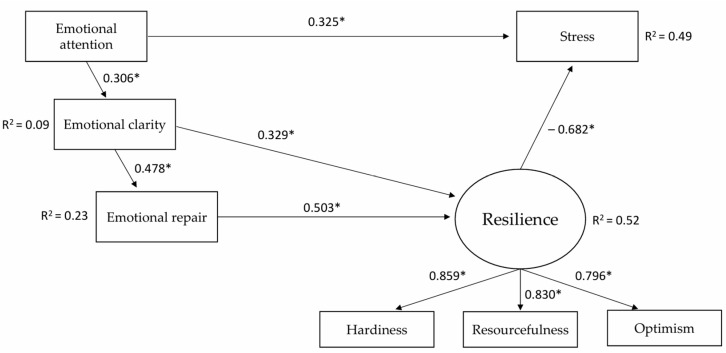
Final model (Model 3). Note. * *p* < 0.001.

To improve model fit, modification indices were considered, while also taking into account their theoretical meaning. First, the modification indexes suggest the inclusion of regression resilience–clarity (mi = 38.878). Once this modification was introduced, the following fit indices were obtained in Model 2: CFI = 0.924; GFI = 0.925, RMSEA = 0.123 and SRMR = 0.072. As can be seen, all the fit indices improved; however, CFI, GFI, and RMSEA still did not reach an acceptable value, and so the modification indices were reconsidered and the influence of EI on burnout was introduced accordingly (mi = 53.075). This relationship can be explained theoretically in that the emotional attention variable, as defined by Mayer and Salovey (1997) in the TMMS-24 measure, refers to the emotional attention that the person pays to their own emotions, and its optimal range denotes that a person has adequate emotional attention, while a high score on this variable indicates an excess of emotional attention, which is itself related to higher stress scores. Once this modification had been introduced, the following fit indices were obtained in Model 3: CFI = 0.980, GFI = 0.967, RMSEA = 0.066, and SRMR = 0.035. The new indices indicated an acceptable model fit, and so we took the latter as the final model (Figure 2). This model explains 49.3% of the variance in stress.

In order to assess the accuracy of the parameter estimated, using the Bollen–Stine method, we applied the bootstrap resampling technique with 5000 bootstrap samples, identifying as significant those regression coefficients for which the 95% interval does not contain the value 0, and setting the random seed to 1.

Table 4 shows all the direct and indirect effects and confidence intervals. The largest direct effect is of resilience on stress (β = −0.682, *p* < 0.001). Emotional repair has a high positive direct effect on resilience (β = 0.503, *p* < 0.001).

Emotional attention has a positive direct effect on emotional clarity (β = 0.306, *p* < 0.001), and emotional clarity has a positive direct effect on emotional repair (β = 0.478, *p* < 0.001).

By contrast, emotional attention has a positive direct effect on stress (β = 0.325, *p* < 0.001) and two indirect negative effects on stress, the first through clarity–repair–resilience (β = −0.050, *p* < 0.001) and the second through clarity–resilience (β = −0.068, *p* < 0.001). Emotional attention also has an indirect positive effect on emotional repair through emotional clarity (β = 0.146, *p* < 0.001). Finally, emotional attention has two indirect positive effects on resilience, the first through clarity–repair (β = −0.050, *p* < 0.001) and the second through emotional clarity (β = −0.068, *p* < 0.001).

Emotional clarity has a direct positive effect on resilience (β = 0.329, *p* < 0.001) and a positive indirect effect through emotional repair (β = 0.240, *p* < 0.001). Likewise, emotional clarity also presents two negative indirect effects on stress, the first through clarity–resilience (β = −0.164, *p* < 0.000), and the second through resilience (β = −0.224, *p* < 0.001).

Finally, emotional repair has an indirect negative effect on stress through resilience (β = −0.343, *p* < 0.001).

## 4. Discussion

This study aimed to test a structural equation model integrating the influence of different dimensions of EI on resilience and stress, and to examine the mediating role of resilience between these dimensions and stress in a sample of university students enrolled in education programs. The final proposed model explained 49.3% of the variance in stress levels, indicating a satisfactory fit and a significant contribution to the understanding of protective factors against stress in this population.

The results largely support the proposed hypotheses. First, Hypothesis 1 was confirmed. This pattern of relationships reproduces the effect proposed in the cascading model by Joseph and Newman (2010), which suggests a hierarchical sequence in the development of emotional abilities; emotional perception precedes emotional understanding, which, in turn, precedes emotional repair.

Hypothesis 2 was partially confirmed by the findings. A direct and positive effect of emotional repair on resilience was observed, consistent with previous studies involving preservice teachers, such as those by García-Martínez et al. (2022) and Rabal-Alonso and González-Romero (2023), as well as Valverde-Janer et al. (2023), who emphasized emotional repair as a key component in strengthening resilience. Among the factors that define resilience, hardiness exhibited the greatest significance, indicating that the ability to face adverse situations with commitment and control plays a substantial role.

However, no direct and negative effect of emotional repair on stress was found; instead, the effect was indirect, suggesting that the ability to repair emotions does not reduce stress directly, but does so by strengthening resilience. Although a direct influence of emotional repair on stress was expected, as proposed in the initial model, the absence of such an effect may indicate that this skill alone does not act as a stress buffer but, rather, needs to be activated through other personal resources, such as resilience, in order to exert a protective impact. Moreover, it is possible that emotional repair, while effective, requires time and training to translate into a noticeable reduction in stress, which would explain why its effect is not immediately or directly observable.

Notably, the emotional attention dimension showed a direct and positive effect on stress, in line with previous research indicating that excessive concern about emotional states, without adequate understanding or regulation, can increase vulnerability to mental health problems. For instance, Yildirim et al. (2023) suggested that a combination of high emotional clarity and low emotional attention was especially protective against issues such as anxiety and depression.

In line with these findings, the results of Sánchez-Zafra et al. (2024) also make sense. Considering the same EI dimensions used in this study, they found that women exhibited higher levels of emotional attention, while men showed higher levels of emotional repair; in addition, women reported higher levels of stress. These data reinforce the idea of a relationship between emotional attention and stress, as those scoring higher in emotional attention—in this case, women—also exhibited higher levels of stress.

Furthermore, Hypothesis 3 was confirmed. The results show that emotional repair positively influences resilience, which, in turn, exerts a direct and negative effect on stress, acting as a mediating mechanism in this relationship. This finding aligns with the results of Sarrionandia et al. (2018), although in their case, an overall EI score was used without breaking it down into its elements. Similarly, Trigueros et al. (2020) identified resilience as a mediating variable between EI (also assessed globally) and stress, although their study specifically addressed academic stress.

Nevertheless, numerous studies, whether by exploring the relationships between the variables included in this study (e.g., Shin & Yeong, 2013) or by incorporating other relevant variables (e.g., Kökçam et al., 2022; Thomas & Zolkoski, 2020), have highlighted that strengthening both EI and resilience can contribute to stress reduction.

Finally, emotional clarity was also found to have a direct and positive effect on resilience, which enriches and expands the model initially proposed. Thus, resilience acted as a mediating variable between two EI dimensions (emotional clarity and emotional repair) and perceived stress, with emotional repair showing a greater influence on resilience. This finding is consistent with those of the study by Meléndez et al. (2019), which found that both emotional clarity and emotional repair significantly and positively predicted resilience in a sample of older adults; by contrast, emotional attention was not a significant predictor. These findings reinforce the idea that not only are emotional abilities related to each other sequentially but also that the specific impacts each dimension has on other variables, such as stress in this case, can differ significantly. In this sense, the unique value of each EI dimension in the ability to cope with adverse situations is highlighted, thus contributing to reducing the negative impact of stress in the university context.

In line with the above, the final model proposed in this study provides empirical evidence on the mechanisms through which EI and resilience influence perceived stress among university students. These findings offer a deeper understanding of how certain emotional abilities, such as emotional clarity and emotional repair, foster the development of resilience and consequently buffer the negative effects of stress. This supports the usefulness of the cascading model in studying EI by emphasizing that not all its dimensions affect the experience of stress in the same way.

The results have important practical and educational implications, particularly within university contexts and, more specifically, in teacher training programs. This relevance stems, on the one hand, from the fact that university students are often exposed to elevated levels of academic, emotional, and career-related stress during a stage of life that is crucial for their overall development. On the other hand, the fact that the sample consists of future education professionals adds a critical dimension, as they are preparing for a career known for its high levels of stress and burnout (Agyapong et al., 2022). In this context, the growing prevalence of mental health issues among young adults underscores the urgency of identifying those protective factors that promote emotional adjustment and prevent the onset of more serious disorders. This study contributes to this need by providing empirical evidence on the moderating role of EI and resilience in stress management. These results can serve as a solid foundation for the design and implementation of targeted intervention programs aimed at developing specific emotional abilities, particularly emotional clarity and emotional repair, which in turn enhance resilience and help buffer the negative effects of stress. Studies such as that by Agyapong et al. (2023) have reviewed evidence demonstrating that training programs have the potential to reduce stress levels.

To be effective, these interventions should be sustained, comprehensive, and embedded in university life from the early stages of higher education. The recommended strategies include the following: (1) emotional abilities workshops, focused on improving emotional repair and clarity; (2) psychoeducational courses integrated into academic curricula to foster EI and resilience; (3) personalized counseling services, especially for students with high emotional attention and stress levels; and (4) peer support networks or mentoring programs to build a stronger sense of community, control, and emotional support.

Furthermore, creating an emotionally supportive educational environment requires institutional commitment. This includes training academic staff in emotional coaching, promoting mental health literacy, and ensuring that psychological services are accessible and destigmatized from the very beginning of students’ university experience.

### Limitations and Future Research Directions

This study also presents some limitations that should be taken into account. First, the sample exclusively comprised students from a faculty of education, particularly preservice teachers. This characteristic limits the generalizability of the findings to other university student profiles.

Therefore, it would be appropriate for future research to expand the sample to students from other disciplines so as to determine whether the results hold true in different academic contexts. Moreover, if the aim is to continue exploring the impacts of the variables included in this study on a relevant population due to their future professional role in school settings, it would be particularly valuable to replicate the proposed model with in-service teachers. This would allow researchers to assess whether the relationships observed between variables remain once professional practice begins, at which point emotional demands and stress levels may be even higher.

Second, future studies are encouraged to adopt a longitudinal approach to examine how these variables evolve.

Third, an additional limitation may stem from the potential bias associated with the exclusive use of self-report measures to assess the variables. To reduce this bias, future research could complement these tools with other types of measurements that enrich the understanding of the processes involved. Along these lines, it would also be relevant to explore other factors that may influence the relationship among the variables analyzed in this study, such as self-confidence, social and family support, psychological well-being, and anxiety; these variables have been linked in previous studies (e.g., Jibaja-Balladares, 2024; Trigueros et al., 2020).

Finally, it would be worthwhile to design and implement interventions that assess the effectiveness of strategies aimed at strengthening both EI and resilience, and at managing stress, whether in the general university population or, and more specifically, in preservice teachers. This group, due to their imminent entry into the professional field, will face high emotional demands, making it especially important to foster emotional abilities in them from the early stages of their training.

## 5. Conclusions

The results of this study provide empirical evidence of the fundamental role that EI and resilience play in perceived stress among university students. The final proposed model, based on a cascading model of EI, enabled a better understanding of how different emotional dimensions are interconnected and how they influence stress both directly and indirectly. In particular, this study confirmed that resilience serves as a key mediating mechanism between emotional clarity, emotional repair, and stress levels, while high emotional attention was found to be directly associated with higher levels of stress.

These findings underscore the potential benefits of promoting the development of emotional abilities while at university, a period of life during which young people are especially vulnerable to mental health issues. Moreover, fostering these abilities in preservice teachers is particularly important, as it not only contributes to their well-being but can also positively impact their future educational practice (Calderón-Palacio, 2023).

## Figures and Tables

**Table 1:** Participants’ gender by age group.

		Female	Male	Total
Age Group	1	193	72	265
	2	35	16	51
	3	18	4	22
Total		246	92	338

Note. Age group 1 = 17–21 years; age group 2 = 22–25 years; age group 3 = 26–50 years.

**Table 2:** Intercorrelation of variables.

Variable	1	2	3	4	5	6	7	M	SD
1. Emotional attention	1							31.30	5.74
2. Emotional clarity	0.306 **	1						28.56	6.34
3. Emotional repair	0.128 *	0.478 **	1					28.59	6.13
4. Hardiness	0.046	0.465 **	0.546 **	1				23.83	5.60
5. Resourcefulness	0.112 *	0.496 **	0.524 **	0.710 **	1			21.35	3.82
6. Optimism	0.101	0.417 **	0.552 **	0.718 **	0.647 **	1		18.12	3.65
7. Stress	0.244 **	−0.353 **	−0.455 **	−0.542 **	−0.558 **	−0.445 **	1	18.60	6.42

Note. N = 338; * *p* < 0.05; ** *p* < 0.001.

**Table 3:** Fit indices.

Index	Model 1	Model 2	Model 3
Comparative fit index (CFI)	0.890	0.924	0.980
Root mean square error of approximation (RMSEA)	0.148	0.123	0.066
Standardized root mean square residual (SRMR)	0.095	0.072	0.035
Goodness of fit index (GFI)	0.897	0.925	0.967
χ^2^ (df)	109.350 (13) *	79.035 (13) **	29.597 (12) *

Note. N = 338; * *p* < 0.05; ** *p* < 0.001.

**Table 4:** Direct and indirect effects, and 95% confidence intervals, of the variables considered in the model.

		Emotional Attention	Emotional Clarity	Emotional Repair	Resilience
Emotional clarity	Direct	0.306 * [0.211–0.400]			
	Indirect	-			
Emotional repair	Direct	-	0.478 * [0.396–0.560]		
	Indirect	0.146 * [0.093–0.199]	-		
Resilience	Direct	-	0.329 * [0.236–0.421]	0.503 * [0.416–0.589]	
	Indirect	0.073 *^a^ [0.044–0.103] 0.100 *^b^ [0.058–0.143]	0.240 * [0.182–0.299]	-	
Stress	Direct	0.325 * [0.248–0.401]	-	-	−0.682 * [−0.749–−0.615]
	Indirect	−0.050 *^c^ [−0.072–−0.028] −0.068 *^d^ [−0.099–−0.038]	−0.164 *^e^ [−0.209–−0.118] −0.224 *^f^ [−0.292–−0.156]	−0.343 * [−0.412–−0.273]	-

Note. N = 338; * *p* < 0.001. ^a^ Indirect effect attention–clarity–repair–resilience; ^b^ Indirect effect attention–clarity–resilience; ^c^ Indirect effect attention–clarity–repair–resilience–stress: ^d^ Indirect effect attention–clarity–resilience–stress; ^e^ Indirect effect clarity–repair–resilience–stress; ^f^ Indirect effect clarity–resilience–stress.

## Data Availability

The raw data supporting the conclusions of this article will be made available by the authors upon request.
